# Color stability of bleached enamel after treatment with CPP-ACP, S-PRG, and sodium fluoride

**DOI:** 10.2340/biid.v13.47065

**Published:** 2026-10-29

**Authors:** André Maciel de Souza, Mayara Zaghi Dal Picolo, Fabiana Mantovani Gomes França, Cecilia Pedroso Turssi, Waldemir Francisco Vieira Junior, Roberta Tarkany Basting

**Affiliations:** Faculdade São Leopoldo Mandic, Campinas, Brazil

**Keywords:** S-PRG, bioactive agents, enamel, anti-erosive dentifrices, dental bleaching

## Abstract

The aim of this study was to evaluate the color stability of bleached teeth after treatment with bioactive materials. Forty bovine teeth underwent four 45-minute bleaching sessions with 35% hydrogen peroxide (Whiteness HP, FGM), followed by application of the respective remineralizing agent after each bleaching session. After the final session, the specimens were randomly assigned (*n* = 10) to one of the following treatments: control (without gel), casein phosphopeptide-amorphous calcium phosphate (CPP-ACP) gel (MI Paste, GC Brasil), S-PRG-based gel (PRG Pro Care Gel, Shofu), or 2% sodium fluoride gel (NaF-Maquira), applied according to the manufacturers’ instructions. The specimens were then immersed in a coffee solution three times daily for 15 minutes over 14 days. Color measurements were performed using a spectrophotometer, evaluating Vita classical shade guide and CIELab* parameters at baseline, after bleaching, and after staining. Data were analyzed using generalized linear mixed models for repeated measures, as well as Friedman, Nemenyi, and Kruskal–Wallis tests (α = 5%). Bleaching significantly increased L* and Whiteness Index for Dentistry (WID) values and reduced a* and b* values (*p* < 0.05). After staining, L* and WID decreased, while a* and b* increased significantly (*p* < 0.05). Compared to baseline, sodium fluoride and S-PRG groups showed higher ΔEab and ΔE00 values than the control (*p* < 0.05). After coffee staining, no differences among groups were found for ΔE*ab, ΔE00, or ΔWID, indicating that none of the bioactive gels altered the susceptibility of bleached enamel to coffee-induced color change.

## Introduction

Tooth bleaching is a conservative procedure widely used in esthetic dentistry, particularly indicated for correcting tooth discoloration, such as intrinsic darkening or yellowing [1, 2]. However, it has been demonstrated that bleached teeth exhibit increased susceptibility to staining when exposed to pigmented beverages, such as coffee, red wine, and tea. This effect is more pronounced in the first weeks following treatment, a period during which enamel may present increased surface porosity, favoring pigment deposition [3, 4].

This increased vulnerability of enamel to pigment deposition may be associated with the pH of certain bleaching agents, which can alter enamel microhardness, surface roughness, and micromorphology [5–7]. Changes in the physical and mechanical properties of enamel during bleaching may decrease microhardness due to mineral loss and a significant increase in surface roughness, even at low concentrations [5, 6]. Furthermore, free radicals derived from hydrogen peroxide (HP) may contribute to these alterations by creating surface irregularities that facilitate the deposition of pigments, compromising post-bleaching color stability and negatively affecting the longevity of esthetic outcomes [8–11].

To reduce the adverse effects of tooth bleaching on enamel, remineralizing approaches with different compositions and mechanisms of action have been proposed as a potential strategy [12, 13]. Bioactive agents and sodium fluoride compounds can interact favorably with the mineral substrate, promoting hydroxyapatite crystal formation due to their remineralizing potential [13, 14].

In this context, casein phosphopeptide-amorphous calcium phosphate (CPP-ACP) compounds have shown potential to promote the formation of calcium and phosphate reservoirs on the tooth surface [15–17]. Under conditions of reduced pH, these compounds facilitate the release of calcium and phosphate ions, maintaining a supersaturated mineral environment [18, 19]. Additionally, CPP-ACP may delay biofilm formation by inhibiting bacterial adhesion and acting as a buffering agent, preventing pH reduction [17].

Surface pre-reacted glass-ionomer (S-PRG) particles are also considered bioactive agents, as they consist of a glass core surrounded by a glass-ionomer phase. These particles can be incorporated into various materials (resins, varnishes, and pastes) and are capable of releasing ions such as aluminum, borate, sodium, silicate, strontium, and fluoride, which contribute to pH elevation and inhibition of demineralization [15, 20, 21]. Ion release may promote the formation of a smoother enamel surface, potentially enhancing resistance to staining [22]. Studies have shown that polishing pastes containing high concentrations of S-PRG particles significantly increase enamel acid resistance and microhardness [21, 23, 24]. Moreover, these ions inhibit enamel demineralization and promote remineralization of carious lesions, suggesting a fundamental role in restoring enamel mineral structure [25].

Sodium fluoride at 2% is also widely used as a remineralizing agent, and its application before, during, and after bleaching has been investigated as a strategy to enhance microhardness and acid resistance of demineralized enamel [26, 27]. Sodium fluoride may reduce enamel demineralization after exposure to bleaching agents and acidic beverages, increasing resistance to acidic challenges, significantly reducing mineral loss, and promoting structural recovery of the substrate [21, 27].

Remineralizing agents have been proposed as a strategy to minimize structural changes in enamel associated with tooth bleaching [6, 13]. Materials containing CPP-ACP, S-PRG particles, or fluoride may interact with the enamel surface through different mechanisms, promoting mineral availability or ion release and potentially influencing enamel properties after bleaching [28]. However, whether the application of these agents affects the subsequent susceptibility of bleached enamel to staining remains unclear. Therefore, the aim of this in vitro study was to evaluate whether the application of CPP-ACP-, S-PRG-, and sodium fluoride-containing materials after in-office bleaching influences the color change of bleached enamel subsequently exposed to coffee.

## Materials and methods

### Experimental design

The experimental units consisted of 40 bovine incisors subjected to four bleaching sessions (35% HP – Whiteness HP, FGM), followed by the application of the respective remineralizing agents after each bleaching session. The specimens were randomly allocated into four experimental groups (*n* = 10) according to the bioactive treatment: Control, no bioactive agent application; CPP-ACP, CPP-ACP-containing paste (MI Paste, GC Corporation, Tokyo, Japan); S-PRG, surface pre-reacted glass-ionomer particle-containing gel (PRG Pro Care Gel, Shofu Dental Corporation, Kyoto, Japan); and NaF, 2% sodium fluoride gel (Fluoride Gel, Maquira, Maringá, PR, Brazil). The remineralizing agents were applied immediately after each bleaching session according to the standardized protocol described below, whereas the control group received no post-bleaching remineralizing treatment. Color was evaluated at three time points: baseline (before bleaching), after completion of the bleaching protocol, and after the 14-day coffee-staining protocol. At each evaluation time point, tooth color was assessed using the VITA classical shade scale and CIELab* coordinates (L*, a*, and b*). The Whiteness Index for Dentistry (WID) was also calculated. Color differences between the evaluation time points were determined using ΔEab, ΔE00, and ΔWID.

### Tooth preparation

Fifty bovine incisors were collected, stored frozen at −18 °C, and thawed at room temperature prior to use. The teeth were cleaned with a scalpel blade to remove periodontal ligament remnants and subjected to prophylaxis using pumice and a Robinson brush. To eliminate irregularities of the buccal surface inherent to bovine teeth, the enamel was superficially ground using 600- and 1200-grit silicon carbide abrasive papers in a water-cooled polishing machine (Aropol 2V, Arotec Ind. e Com., São Paulo, SP, Brazil). An adhesive label (5 × 5 mm) was placed at the center of the buccal surface, and the entire crown was isolated with nail polish (Impala, Guarulhos, SP Brazil). The unpainted enamel area (middle third of the crown) was submitted to treatments, staining, and color analyses. Initial color was evaluated by a spectrophotometer (VITA Easyshade^®^ Advance, Vita, Germany) with controlled illumination conditions, for with preliminary classification based on the Vita classical shade guide. Teeth that were excessively light or excessively dark were excluded, and 40 teeth with shades darker than A2 were selected to ensure homogeneous distribution among the four experimental groups (*n* = 10). The teeth were embedded by their root portion up to the cementoenamel junction using polyester resin (Maxi Rubber, Campinas, SP, Brazil), maintaining the long axis of the tooth perpendicular to the horizontal plane. After resin polymerization, the specimens were stored immersed for 7 days in artificial saliva (5 mM Ca, 0.9 mM P, 150 mM KCl, 0.05 μg F/mL in 0.1 mol/L Tris; pH = 7.0) [29].

### Bleaching treatment and application of remineralizing agents

The bleaching agent and the remineralizing agents used in the present study are described in Table 1. The pH of the gels was measured in triplicate using a digital pH meter (pH meter TEC-2, Tecnal, Piracicaba, São Paulo, Brazil). The bleaching gel containing 35% HP (Whiteness HP, FGM) was prepared immediately prior to each application by mixing one drop of the thickening agent with three drops of the HP liquid phase, followed by manual mixing for 15 seconds, according to the manufacturer’s recommendations. All samples received application of the bleaching agent on the buccal surface of the bovine incisors, using a volume of 0.03 mL. The product was evenly spread over the entire delimited area and remained in contact for 45 minutes. At the end of application period, the gel was removed with gauze, and the surface was rinsed with water for 10 seconds. Subsequently, a thin and uniform layer of the remineralizing agents was applied to the respective experimental groups according to the assigned treatments (CPP-ACP, PRG, or NaF), using a hypodermic syringe and following a standardized protocol based on the manufacturers’ instructions and left undisturbed for 4 minutes. The control group did not receive any remineralizing agent. Removal was performed using a microbrush, followed by rinsing with distilled water for 10 seconds. The specimens were then returned to artificial saliva, which was renewed every 2 days. This protocol was carried out over four clinical sessions, with a 1-week interval between sessions.

**Table 1:** Specification of the bleaching agent and bioactive dentifrices and their respective compositions.

Materials (manufacturer/batch number)	Composition/pH
*HP:* 35% HP (Whiteness HP, FGM, Joinville-SC, Brasil)/Batch 90124	35% HP, thickeners, dyes, glycol, inorganic fillers, and deionized water/pH = 5.82
*CPP-ACP:* CPP-ACP Gel (casein phosphopeptide–amorphous calcium phosphate) (MI Paste, CG Corporation, São Paulo, SP, Brasil)/Batch 220428ª	Purified water, glycerol, casein phosphopeptide–amorphous calcium phosphate, D-sorbitol, sodium carboxymethylcellulose (CMC-Na), propylene glycol, silicon dioxide, titanium dioxide, xylitol, phosphoric acid, flavoring agent, sodium saccharin, ethyl p-hydroxybenzoate, propyl p-hydroxybenzoate, and butyl p-hydroxybenzoate/pH = 6.96
*PRG:* S-PRG Gel (PRG Pro Care Gel, Shofu Dental Corporation, Kioto, Japão)/Lote052201	Hydrated silica, S-PRG particles, glycerol, sodium carboxymethylcellulose, sorbitol, sodium dodecyl sulfate, and mint flavoring/pH = 6.85
*NaF:* 2% sodium fluoride gel (Flúor Gel, Maquira, Maringá, PR, Brasil)/Lote 165222	2% sodium fluoride, sodium saccharin, propylene glycol, methylparaben, colorant, flavoring agent, excipients, and purified water/pH = 6.78

CPP-ACP: casein phosphopeptide-amorphous calcium phosphate; S-PRG: surface pre-reacted glass-ionomer; NaF: sodium fluoride gel; HP: hydrogen peroxide.

### Coffee staining protocol

The staining protocol was based on the studies of Araújo et al. and Kobayashi et al. [30, 31], simulating coffee staining over a 14-day period. Accordingly, 24 hours after completion of the fourth bleaching session, the teeth were immersed in 5 mL of coffee solution (Nescafé Tradição, Araras, SP, Brazil). The solution was prepared by dissolving 16 g of coffee in 200 mL of distilled water. Immersions were performed three times per day, with 3-hour intervals between them. Each immersion lasted 15 minutes at 37 °C for 14 days. After each immersion, the specimens were rinsed with distilled water for 2 minutes and then immersed in artificial saliva between staining cycles. All immersions were conducted at room temperature. The pH of the coffee solution was measured prior to each immersion using a pH meter, with an average value of 4.75.

### Color evaluation

Color measurements were performed using a spectrophotometer (VITA Easyshade^®^ Advance, VITA Zahnfabrik, Germany) at three time points: baseline (before bleaching), after completion of the bleaching protocol, and after the 14-day coffee-staining protocol. For the analyses, the dental specimens were positioned inside a chamber with a white background to standardize lighting conditions. The spectrophotometer was calibrated prior to the color measurements using its automatic calibration function. Tooth color was assessed with the measuring tip positioned perpendicular to and fully in contact with the enamel surface. Each measurement was performed three times consecutively. Color change (ΔEab) was calculated for each group and evaluation period using the formula [32]: ΔEab = √((ΔL*)² + (Δa*)² + (Δb*)²).

The perceptibility and acceptability thresholds for ΔEab were set at 1.2 and 2.7, respectively [32, 33]. Color change was also assessed using the CIEDE2000 formula (ΔE00). ΔE00 values of 0.8 and 1.8 were adopted as the perceptibility and acceptability thresholds, respectively [33]. Color change was additionally evaluated using the WID; Δ values considering the same time points above were calculated for (ΔWID), based on the CIELAB system: = 0.55L* − 2.32a* − 1.100b*. The 50:50% perceptibility threshold for the whiteness index change (WPT) was considered 0.61 ΔWID units.

Threshold values for ΔWID were set at 0.72 for perceptibility and 2.60 for acceptability [34]. Tooth color was determined according to the VITA classical shade guide. Subsequently, the VITA classical scale was converted into numerical values based on previously established literature, ranging from 1 (B1) to 16 (C4) in descending order of value: B1, A1, B2, D2, A2, C1, C2, D4, A3, D3, B3, A3.5, B4, C3, A4, and C4. Lower numerical values correspond to lighter tooth shades [35, 36].

### Enamel surface micromorphology

The enamel surface micromorphology after the treatments was evaluated using scanning electron microscopy (SEM). Three specimens from each group were dried and dehydrated in a desiccator for 12 hours. The enamel surfaces were coated with a gold layer using vacuum sputtering and examined under a scanning electron microscope (Leo 440i, LEO Electron Microscopy/Oxford, Cambridge, England) at 5000× magnification and 20 kV accelerating voltage, evaluating the presence of porosities and surface irregularities.

### Statistical analysis

All statistical analyses were performed using R software (R Core Team, 2024), with the significance level set at 5%. Descriptive and exploratory analyses were initially conducted. During the exploratory analysis, the distribution of errors and the presence of potential outliers were examined using boxplots and dot plots. Residual normality was also assessed using the Shapiro–Wilk test, which indicated deviation from a normal distribution. Therefore, the assumption of normally distributed residuals required for classical analysis of variance was not met. Generalized linear mixed models (GLMMs) for repeated measures over time were subsequently fitted to analyze L* and b* values, assuming a gamma distribution with a logarithmic link function. Group, time, and the group × time interaction were included as fixed effects. Because the same teeth were evaluated at three time points, tooth was included as a random effect by specifying a random intercept to account for the correlation among repeated measurements within the same specimen. Multiple comparisons were performed using Wald tests with Bonferroni correction. VITA classical shade scores, a* values, and WID values did not adequately fit the evaluated statistical distributions and were therefore analyzed using nonparametric methods. Comparisons among evaluation time points, accounting for the dependence among repeated measurements obtained from the same specimen, were performed using the Friedman test, followed by the Nemenyi test for multiple comparisons. Comparisons among experimental groups were performed using the Kruskal–Wallis test, as different specimens were evaluated in each group. ΔEab and ΔE00 values were analyzed separately for each evaluation interval using generalized linear models (GLMs) assuming a gamma distribution with a logarithmic link function, with group included as a fixed effect. When a significant group effect was detected, multiple comparisons were performed using Wald tests with Bonferroni correction. Comparisons among groups for ΔWID were also performed separately for each evaluation interval using the nonparametric Kruskal–Wallis test.

## Results

No significant differences were observed among the groups regarding the Vita classical shade score (Table 2). In all groups, there was a significant decrease in the score after bleaching treatment and a significant increase after coffee staining (*p* < 0.05). Similarly, no significant differences were found among the groups for the L value, as shown in Table 3. In all four groups, the L value increased significantly after bleaching treatment and decreased significantly after coffee staining (*p* < 0.05). Likewise, regardless of the group, both a and b values decreased significantly after bleaching treatment and increased after staining (*p* < 0.05), as presented in Tables 4 and 5. As shown in Table 6, the WID increased after bleaching treatment and decreased after staining, with no significant differences among the groups (*p* > 0.05). Regarding post-bleaching color stability, no significant differences among the experimental groups were observed for ΔEab (*p* = 0.9701), ΔE00 (*p* = 0.9559), or ΔWID (*p* = 0.9385) when color changes were calculated from the post-bleaching to the post-coffee-staining time point (Tables 7 and 8). When the overall color change from baseline to post-staining was considered, significant differences among groups were observed for ΔEab (*p* = 0.0142) and ΔE00 (*p* = 0.0438), whereas no significant difference was detected for ΔWID (*p* = 0.9570).

**Table 2:** Median (minimum–maximum) values of the Vita classical shade scale as a function of the bioactive gel and evaluation time.

Bioactive agent	Evaluation time			*p*
	Baseline	After bleaching	After coffee staining	
Control	9.00 (9.00; 11.00)^Aa^	2.00 (1.00; 2.00)^Ba^	9.00 (3.00; 15.00)^Aa^	0.0004
CPP-ACP	9.00 (9.00; 11.00)^Aa^	1.50 (1.00; 2.00)^Ba^	7.00 (2.00; 15.00)^Aa^	0.0008
PRG	9.00 (9.00; 11.00)^Aa^	2.00 (1.00; 2.00)^Ba^	9.00 (3.00; 15.00)^Aa^	0.0005
NaF	9.00 (9.00; 11.00)^Aa^	2.00 (1.00; 2.00)^Ba^	7.00 (2.00; 16.00)^Aa^	0.0015
*p*	0.9443	0.2940	0.9700	

Different letters (uppercase horizontally and lowercase vertically) indicate statistically significant differences (*p* < 0.05). CPP-ACP: casein phosphopeptide-amorphous calcium phosphate; PRG: pre-reacted glass-ionomer; NaF: sodium fluoride gel.

**Table 3:** Mean (standard deviation) of L* as a function of the bioactive gel and evaluation time.

Bioactive agent	Evaluation time		
	Baseline	After bleaching	After coffee staining
Control	89.72 (1.35)^Ba^	94.42 (3.10)^Aa^	86.76 (4.19)^Ca^
CPP-ACP	89.61 (1.82)^Ba^	94.77 (2.40)^Aa^	84.62 (4.55)^Ca^
PRG	87.92 (2.27)^Ba^	93.44 (2.56)^Aa^	85.64 (6.35)^Ca^
NaF	87.66 (2.97)^Ba^	93.75 (2.37)^Aa^	85.22 (8.41)^Ca^

*p* (gel) = 0.4871; *p* (time) < 0.0001; *p* (interaction) = 0.8168. Different letters (uppercase horizontally and lowercase vertically) indicate statistically significant differences (*p* ≤ 0.05). CPP-ACP: casein phosphopeptide-amorphous calcium phosphate; PRG: pre-reacted glass-ionomer; NaF: sodium fluoride gel.

**Table 4:** Median (minimum–maximum) of a* as a function of the bioactive gel and evaluation time.

Bioactive agent	Evaluation time			*p*
	Baseline	After bleaching	After coffee staining	
Control	1.05 (0.20; 2.40)^Aa^	–1.20 (–2.30; –0.10)^Ba^	1.60 (0.10; 6.80)^Aa^	0.0006
CPP-ACP	0.35 (–0.60; 1.20)^Aa^	–1.20 (–1.80; –0.20)^Ba^	1.75 (0.30; 6.00)^Aa^	0.0001
PRG	0.30 (–0.60; 9.90)^Aa^	–1.05 (–1.90; 1.40)^Ba^	2.05 (–0.10; 31.90)^Aa^	0.0018
NaF	0.75 (–0.30; 1.70)^Aa^	–1.15 (–1.90; 0.10)^Ba^	1.60 (–1.50; 12.20)^Aa^	0.0006
*p*	0.2133	0.7489	0.9826	

Different letters (uppercase horizontally and lowercase vertically) indicate statistically significant differences (*p* ≤ 0.05). CPP-ACP: casein phosphopeptide-amorphous calcium phosphate; PRG: pre-reacted glass-ionomer; NaF: sodium fluoride gel.

**Table 5:** Mean (standard deviation) of b* as a function of the bioactive gel and evaluation time.

Bioactive agent	Evaluation time		
	Baseline	After bleaching	After coffee staining
Control	26.40 (2.63)^Aa^	12.84 (5.02)^Ba^	25.41 (4.31)^Aa^
CPP-ACP	26.61 (1.53)^Aa^	14.96 (2.69)^Ba^	24.67 (4.54)^Aa^
PRG	26.93 (3.00)^Aa^	16.83 (2.25)^Ba^	26.38 (3.63)^Aa^
NaF	25.61 (1.63)^Aa^	16.90 (3.22)^Ba^	25.50 (9.19)^Aa^

*p* (gel) = 0.1999; *p* (time) < 0.0001; *p* (interaction) = 0.3308. Different letters (uppercase horizontally and lowercase vertically) indicate statistically significant differences (*p* ≤ 0.05). CPP-ACP: casein phosphopeptide-amorphous calcium phosphate; PRG: pre-reacted glass-ionomer; NaF: sodium fluoride gel.

**Table 6:** Median (minimum–maximum) of the Whiteness Index for Dentistry as a function of the bioactive gel and evaluation time.

Bioactive agent	Evaluation time			*p*
	Baseline	After bleaching	After coffee staining	
Control	14.34 (7.70; 20.64)^Ba^	37.14 (28.10; 45.87)^Aa^	13.11 (–12.62; 26.54)^Ba^	0.0006
CPP-ACP	16.25 (8.57; 19.97)^Ba^	33.99 (29.00; 44.39)^Aa^	12.17 (–9.23; 24.57)^Ba^	0.0005
PRG	13.64 (–5.79; 21.16)^Ba^	32.86 (23.21; 39.62)^Aa^	7.43 (–57.17; 21.39)^Ba^	0.0006
NaF	15.18 (9.63; 19.63)^Ba^	33.13 (21.92; 39.34)^Aa^	13.19 (–40.51; 36.38)^Ba^	0.0022
*p*	0.6249	0.2339	0.8785	

Different letters (uppercase horizontally and lowercase vertically) indicate statistically significant differences (*p* ≤ 0.05). CPP-ACP: casein phosphopeptide-amorphous calcium phosphate; PRG: pre-reacted glass-ionomer; NaF: sodium fluoride gel.

**Table 7:** Mean (standard deviation) of ΔEab and ΔE00 as a function of the bioactive gel.

Parameter	Bioactive agent	Time interval		
		After bleaching-baseline	After coffee staining-baseline	After coffee staining-after bleaching
ΔE_ab_	Control	14.85 (4.28)^a^	4.94 (4.70)^b^	15.72 (7.33)^a^
	CPP-ACP	13.01 (3.14)^a^	7.51 (3.51)^ab^	14.74 (5.99)^a^
	PRG	12.67 (4.50)^a^	11.30 (8.39)^a^	15.21 (8.12)^a^
	NaF	11.16 (4.51)^a^	10.78 (6.02)^a^	13.90 (11.26)^a^
	*p*	0.2471	0.0142	0.9701
ΔE_00_	Control	8.59 (2.78)^a^	3.09 (3.14)^b^	9.51 (4.63)^a^
	CPP-ACP	7.20 (1.96)^a^	4.72 (2.59)^ab^	9.17 (3.71)^a^
	PRG	7.36 (2.77)^a^	7.19 (6.47)^a^	9.33 (5.70)^a^
	NaF	6.49 (2.63)^a^	6.26 (3.99)^a^	8.30 (6.68)^a^
	*p*	0.3012	0.0438	0.9559

Different letters within the same column indicate statistically significant differences among groups (*p* ≤ 0.05) for each parameter. CPP-ACP: casein phosphopeptide-amorphous calcium phosphate; PRG: pre-reacted glass-ionomer; NaF: sodium fluoride gel.

**Table 8:** Median (minimum–maximum) of ΔWID as a function of the bioactive gel.

Bioactive agent	Time interval		
	After bleaching-baseline	After coffee staining-baseline	After coffee staining-after bleaching
Control	22.88 (15.53; 28.71)^a^	–0.07 (–28.64; 6.85)^a^	–22.07 (–55.70; –13.68)^a^
CPP-ACP	18.54 (12.07; 29.68)^a^	–3.87 (–26.41; 6.99)^a^	–24.02 (–45.14; –7.48)^a^
PRG	16.86 (9.23; 37.42)^a^	–2.72 (–77.28; 16.13)^a^	–23.41 (–90.16; –12.53)^a^
NaF	15.11 (8.32; 27.85)^a^	–2.90 (–50.46; 18.92)^a^	–19.70 (–65.91; 3.31)^a^
*p*	0.2102	0.9570	0.9385

Identical letters within the same column indicate no statistically significant differences among groups (*p* > 0.05). WID: Whiteness Index for Dentistry; CPP-ACP: casein phosphopeptide-amorphous calcium phosphate; PRG: pre-reacted glass-ionomer; NaF: sodium fluoride gel.

SEM images revealed scratches and linear surface features consistent with the grinding and polishing procedures used during specimen preparation. Qualitative examination of the images did not reveal obvious differences in enamel surface micromorphology among the experimental groups under the SEM conditions used in this study (Figure 1).

**Figure 1:**
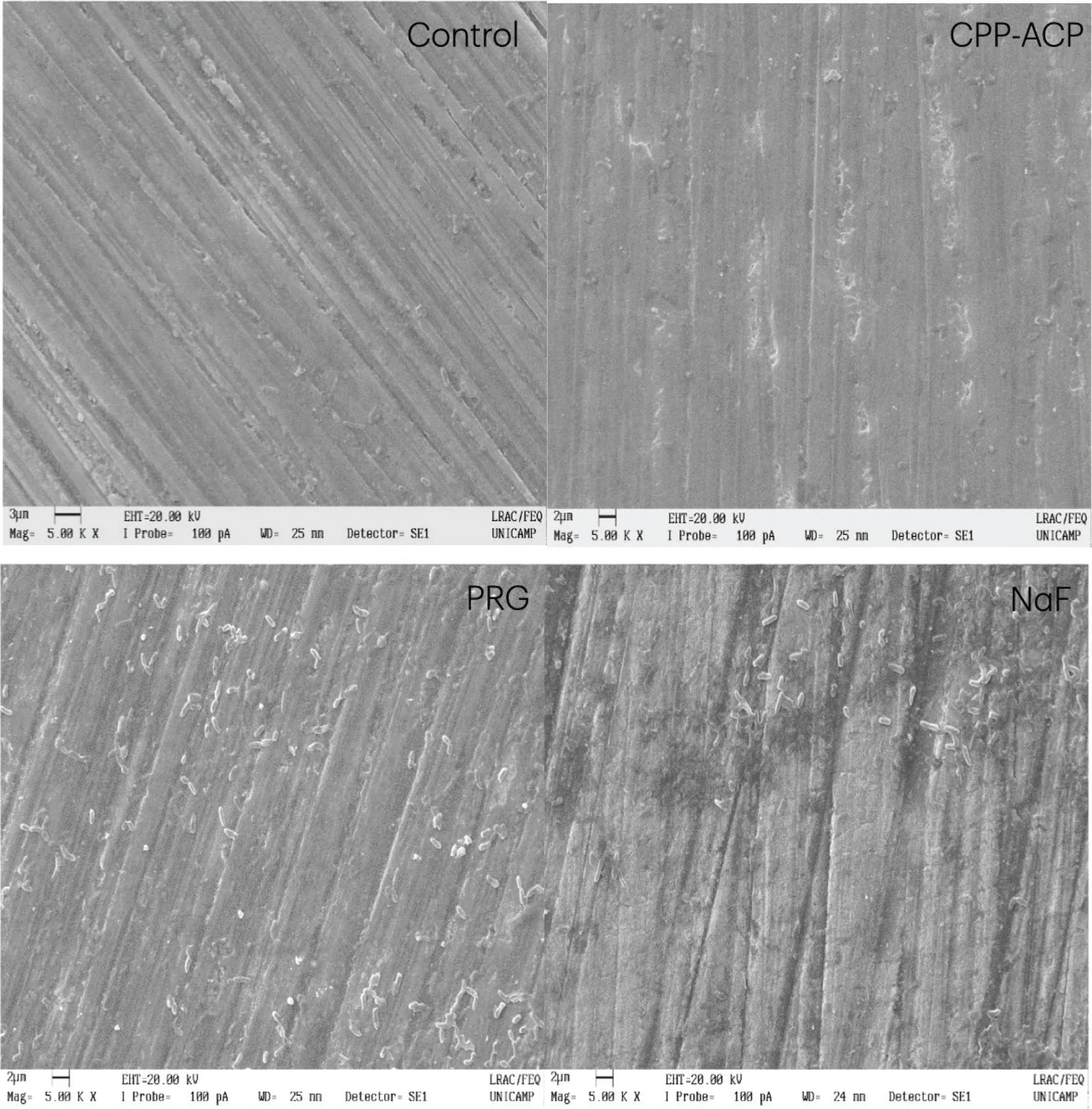
Surface micromorphology images of enamel subjected to different treatments with bioactive gels. CPP-ACP: casein phosphopeptide-amorphous calcium phosphate; PRG: pre-reacted glass-ionomer; NaF: sodium fluoride gel.

## Discussion

Tooth bleaching is widely recognized as an effective procedure for modifying tooth color, resulting in significant esthetic improvement and meeting the growing patient demand for whiter and more harmonious smiles. In this context, the findings of the present study demonstrated that bleaching treatment with 35% HP, using an in-office application protocol, produced a significant whitening effect. Statistically significant changes were observed in all evaluated colorimetric parameters (L*, a*, b*, ΔE_ab_, ΔE_00_, and ΔWID), as well as a reduction of up to seven units in the Vita classical shade scale.

Systematic reviews and meta-analyses have demonstrated the clinical efficacy of HP and carbamide peroxide-based bleaching agents in promoting clinically significant changes in tooth color [37, 38]. The mechanism of action of bleaching is based on the degradation of chromophores, which are complex molecules responsible for dental pigmentation. HP decomposes and releases low molecular weight free radicals that penetrate into the enamel and dentin, leading to oxidation and breakdown of long molecular chains into smaller, less visible molecules, resulting in lighter teeth [39]. This degradation process is dependent on gel concentration, application time, and product composition. The color changes observed, particularly in the ΔE_00_ parameter, exceeded perceptibility and acceptability thresholds, confirming that the bleaching protocol used in the present study yields clinically perceptible and acceptable esthetic outcomes.

However, some bleaching agents may induce alterations in the dental substrate, including increased surface roughness and reduced enamel microhardness [39, 40], thereby facilitating the deposition of exogenous pigments and contributing to dental discoloration [41, 42]. The use of bioactive agents containing calcium, phosphate, fluoride, or bioactive glass particles (S-PRG) during bleaching may help minimize structural changes, promote enamel remineralization, and reduce post-bleaching staining [43, 44].

In this regard, it can be observed that none of the remineralizing agents applied during bleaching in the present study interfered with the bleaching outcome, both in terms of individual color parameters and overall color change (ΔEab, ΔE00, and ΔWID) between baseline and post-bleaching time points. Two of the evaluated remineralizing agents were colorless gels, whereas the CPP-ACP–based product (MI Paste) exhibited a white coloration due to the presence of titanium dioxide in its formulation. Although no analyses of enamel microhardness or surface roughness were performed to assess potential substrate alterations caused by the bleaching agent, neither the bleaching gel alone (control group) nor the use of remineralizing agents resulted in observable micromorphological changes in the enamel surface. It is noteworthy that the evaluated remineralizing agents exhibited near-neutral pH values (6.96, 6.85, and 6.78 for CPP-ACP, PRG, and NaF, respectively), which would not be expected to induce micromorphological alterations or changes in the mineral content of enamel.

Coffee staining has been widely used as an experimental model to evaluate post-bleaching color stability, as it contains low molecular weight chromogenic pigments capable of penetrating the dental structure. Additionally, coffee presents an acidic pH (approximately 4.7), which confers erosive potential and further promotes pigment incorporation into the bleached enamel surface [45–47].

In the present study, none of the evaluated remineralizing agents significantly affected the color change induced by coffee after bleaching. When the post-bleaching condition was used as the reference, no significant differences among the control, CPP-ACP, S-PRG, and NaF groups were detected for ΔEab, ΔE00, or ΔWID. These findings indicate that, under the experimental conditions evaluated, the application of the tested remineralizing agents neither reduced nor increased the susceptibility of bleached enamel to coffee-induced color change. Although significant differences among groups were observed for ΔEab and ΔE00 when the post-staining condition was compared with baseline, this interval encompasses both the bleaching-induced color change and the subsequent effect of coffee exposure. Therefore, these differences could not be attributed specifically to staining susceptibility. Similarly, because the CPP-ACP group did not differ significantly from either the control or the S-PRG and NaF groups in the post-bleaching to post-staining comparison, the present findings do not support a specific protective effect of CPP-ACP on post-bleaching color stability.

The absence of statistically significant differences among groups after coffee staining for the evaluated color parameters (Vita classical, L*, a*, b*, and WID) suggests that the remineralizing agents did not influence overall color stability. However, the greater color change (ΔEab and ΔE00) observed between baseline and post-staining time points in the S-PRG and NaF groups may indicate increased susceptibility to staining, requiring further investigation. The S-PRG particles in the paste, as well as the deposition of calcium fluoride potentially promoted by NaF application, may have altered enamel surface roughness, facilitating the incorporation of coffee pigments. This interpretation is supported by Dionysopoulos [47], who reported that fluoride treatments following bleaching significantly increased enamel surface microhardness, which may influence pigment uptake.

The observation that CPP-ACP may have contributed to maintaining color stability is consistent with the findings of Poggio [48], who demonstrated the protective effect of CPP-ACP–containing pastes against acid erosion, reinforcing its role in preserving enamel integrity. However, when evaluating color change using the ΔWID parameter, no differences were observed among groups. In this context, staining in the treated groups (PRG and NaF) exhibited a crucial characteristic: color change was predominantly concentrated in chromaticity (variations in the a* and b* axes). Clinically, this indicates that the primary perceptible change occurred in tooth hue, with pigment absorption leading to a more yellowish or reddish appearance, rather than a true darkening (loss of luminosity, represented by L*). This distinction explains the greater sensitivity of the ΔE00 (CIEDE2000) parameter for analyzing the present data. In contrast, the ΔWID index proved to be limited in this study, as it assigns disproportionately greater weight to luminosity (L*), thereby minimizing the impact of chromatic variations. Since coffee staining did not result in significant darkening (minimal change in L*), ΔWID failed to detect chromatic variations that, although clinically relevant, did not involve loss of brightness.

The findings of this study highlight the complexity of the interactions among bleaching agents, remineralizing agents, and staining susceptibility. The use of remineralizing agents not only promotes mineral deposition and recovery of demineralized enamel hardness [49] but also presents specific clinical challenges [50, 51]. According to Lee [29], the interaction between these systems and bleaching agents may compromise the color stability of bioactive restorative materials due to ion release dynamics that alter surface properties and the chromatic perception of dental tissues and restorative materials.

The need for improved strategies to maintain post-bleaching color stability is evident. Future studies should focus on elucidating the mechanisms by which remineralizing agents influence enamel microstructure and pigment adhesion. The evaluation of different application protocols and the exploration of combinations of protective agents may contribute to the development of more effective approaches for preventing staining.

## Conclusion

Under the conditions of this in vitro study, none of the evaluated remineralizing agents reduced coffee-induced color change after bleaching. No significant differences among groups were detected for ΔE*ab, ΔE00, or ΔWID calculated from the post-bleaching to the post-staining time point. Therefore, the data support neither a protective effect nor an increased staining susceptibility attributable to any of the tested agents.
